# Strontium-Decorated Ag_2_O Nanoparticles Obtained via Green Synthesis/Polyvinyl Alcohol Films for Wound Dressing Applications

**DOI:** 10.3390/ma18153568

**Published:** 2025-07-30

**Authors:** Vanita Ghatti, Sharanappa Chapi, Yogesh Kumar Kumarswamy, Nagaraj Nandihalli, Deepak R. Kasai

**Affiliations:** 1 Department of Chemistry, Faculty of Engineering and Technology, Jain University, Jakkasandra 562112, Karnataka, India; vanitaghatti@gmail.com; 2 Department of Physics, B.M.S. College of Engineering, Bengaluru 560019, Karnataka, India; 3 Department of Sciences, Alliance School of Sciences, Alliance University, Central Campus, Anekal, Bengaluru 562106, Karnataka, India; yogeshkk3@gmail.com; 4 Critical Materials Innovation Hub, Ames National Laboratory, U.S. Department of Energy, Iowa State University, Ames, IA 50011, USA; nnandiha@uwaterloo.ca

**Keywords:** PVA, Sr-coated Ag_2_O nanoparticles, antimicrobial activity, haemolysis, cytotoxicity, wound healing

## Abstract

This study involved the fabrication of poly (vinyl alcohol) (PVA) nanocomposite films using the solution-casting process, which incorporated strontium-coated silver oxide (Sr-Ag_2_O) nanoparticles generated by a plant-extract assisted method. Various characterization techniques, such as XRD, SEM, TEM, UV, and FTIR, showed the formation and uniform distribution of Sr-Ag_2_O nanoparticles in the PVA film, which are biocompatible nanocomposite films. The presence of hydroxyl groups leads to appreciable mixing and interaction between the Sr-Ag_2_O nanoparticles and the PVA polymer. Mechanical and thermal results suggest enhanced tensile strength and increased thermal stability. In addition, the sample of PVA/Sr-Ag_2_O (1.94/0.06 wt. ratio) nanocomposite film showed decreased hydrophilicity, lower hemolysis, non-toxicity, and appreciable cell migration activity, with nearly 19.95% cell migration compared to the standard drug, and the presence of Sr-Ag_2_O nanoparticles favored the adhesion and spreading of cells, which triggered the reduction in the gaps. These research findings suggest that PVA/Sr-Ag_2_O nanocomposite films with good mechanical, antimicrobial, non-toxic, and biocompatible properties could be applied in biological wound-healing applications.

## 1. Introduction

The skin is the body’s first line of protection, yet it is also the part of the body that is most vulnerable to injury from burns, trauma, and surgery. The skin serves as a defensive barrier that is essential for avoiding wounds. Infection of a wound occurs when bacteria and other microbes settle into the area, which can slow healing or even cause the lesion to worsen [1,2]. Bacteria, which can enter the body through the skin, other bodily components, or even external factors, cause the majority of wound infections. Each healthy layer of skin, epidermis, dermis, and fatty subcutaneous layer works together to form a protective barrier [3]. However, once the barrier has been penetrated, bacteria disrupt the outer epidermal barrier, denature proteins and lipids, and create an ideal environment for their growth [4,5]. The outcome is an infection that sets off a chain reaction within the immune system, which in turn causes inflammation and slows down the healing process [6]. Infected wounds often recover on their own, but severe wounds that are not treated properly or at all might lead to serious complications or even death. Therefore, the objective of wound care is to prevent or eliminate harmful germs while facilitating wound healing [7].

Traditional wound care has relied on the use of dressings as a physical barrier to prevent the spread of infection [8]. Over time, more sophisticated dressings containing antibiotics or other antiseptic chemicals have supplanted older, nondrug-based dressings. Wound coverings commonly incorporate the following classes of antibiotics: quinolones, cephalosporins, aminoglycosides, and tetracyclines [9,10]. These medications disrupt bacterial metabolism by changing protein and nucleic acid production or by weakening the bacterial cell wall, which in turn causes metabolic abnormalities. Researchers have explored novel wound-healing nano-based materials that utilize nanoparticles to target harmful bacteria, thereby slowing the spread of antibiotic-resistant strains. The perfect therapeutic wound covering would not cling to wound surfaces, would be mechanically strong, would be flexible, and would absorb wound exudate [11].

The term “green synthesis” has recently emerged in the field of nanotechnology to describe the process of making nanoparticles (NPs) without the use of hazardous materials that produce undesirable waste products [12]. To rephrase this, a sustainable approach enables NPs to be synthesized in a manner that is safe for both humans and the environment, without compromising either [13,14]. Materials of the nanoscale range, such as silver oxide, titanium oxide, zinc oxide, strontium oxide, graphene oxide, and CNTs, are the building blocks of nanotechnology, a broad field of technology that has just emerged. Both medicine and farming have undergone significant changes with the introduction of polymers and nanomaterials, which have enabled more precise medication delivery in healthcare and enhanced crop production and soil quality in agriculture [15,16,17,18,19]. Different classes of nanocomposite films with reinforced properties are created by doping polymer networks with nanoparticles. Scientists have spent years refining nanotechnology, which enables them to develop nanomaterials using biomolecules derived from living organisms, particularly plants. These biomolecules include proteins, lipids, secondary metabolites, and metals [20].

Due to its numerous beneficial biomedical characteristics, the Fabaceae family legume Lablab purpureus (LP) has gained popularity recently. It is one of the most cultivated species of legumes. Traditional medical systems, such as Ayurveda and Chinese medicine, extensively utilize plant bioactives for the treatment of various illnesses, including cholera, food poisoning, uterine inflammation, diarrhea, and phlegmatic diseases [21]. Extracts from fresh LP were found to contain a wide variety of chemicals, including sugar, alcohols, steroids, tannins, flavonoids, saponins, coumarins, terpenoids, pigments, and glycosides, according to phytochemical research [22,23,24]. As the LP peel extract contains natural reducing agents, such as polyphenols, flavonoids, and alkaloids, these moieties can effectively reduce metal ions to nanoparticles. Different nanocomposite films with reinforced properties are created by doping the polymer network with nanoparticles. For example, to regulate the release of medicines and enhance their antibacterial action, carboxymethyl chitosan–polyvinyl alcohol has been modified using silver nanoparticles [25]. Adding iron oxide nanoparticles to polyethylene glycol improved its antimicrobial capabilities [26]. The hydrogels composed of cerium-incorporated chitosan and PVA were investigated by Kalantari et al. [27] for their efficacy as a wound dressing. Potentially effective antibacterial, radical scavenging, and wound-healing hydrogels decorated with CuO are based on carboxymethyl starch [28].

In the medical field, PVA is one example of a synthetic polymer and it has a well-known semicrystalline structure. PVA is biodegradable, biocompatible, and highly soluble in water, and exhibits chemical resistance and low carbohydrate adsorption [29]. It is entirely compatible with other biopolymers and polymers that exhibit hydrophilic characteristics, and it forms films [30]. PVA’s many medical applications include wound dressings, soft contact lenses, eye drops, embolic filters, and synthetic meniscus and cartilage [29,31]. Strontium is a trace element that shares characteristics with two other minerals that are essential for human health: calcium and magnesium. The dental, bone, and tissue engineering fields rely on strontium for its valuable properties. Recently, more people have become interested in strontium and strontium-conjugated nanoparticles due to their numerous known applications in fields such as bio-imaging, cancer treatment, effective drug delivery, and bone engineering, among others. These components are crucial because they govern the pace of degradation, promote drug release, enhance wound healing, and improve mechanical qualities for tenacity and endurance, thereby enhancing the biological response [32]. Adding Sr NPs to various polymers and metal alloys greatly enhances their mechanical and biological characteristics [33].

The antimicrobial and wound-healing properties of strontium oxide nanoparticles (SrO_2_) make them promising candidates for use in the treatment of diabetes and other conditions [34,35]. According to Joy et al. [36], a polycaprolactone nanocomposite that was made using GO/SrO_2_ showed promising antibacterial and cytotoxic properties. A plant extract from the Pedalium murex plant was used to make mixed strontium cerium oxide (SrCeO_3_) nanoparticles. These nanoparticles exhibited antibacterial activity against both Gram-positive and Gram-negative bacteria. The manufacturing process required ionic liquid [37]. This study aims to develop a more efficient method for synthesizing strontium (Sr)-coated silver oxide (Ag_2_O) nanoparticles using LP peel extract via a hydrothermal approach. The influence of different concentrations of Sr-Ag_2_O nanoparticles on PVA film and their physicochemical properties towards wound-healing applications were investigated. The fabricated nanocomposite films were examined using UV–visible spectroscopy, FTIR, a universal testing machine, SEM, TEM, X-ray diffraction, and thermogravimetric analysis. Additionally, antimicrobial toxicity, hemocompatibility, and wound-healing scratch assays were extended to the prepared nanocomposite films.

## 2. Materials and Methods

### 2.1. Materials

Poly(vinyl acetate) (PVAc) of laboratory grade (M.W. of ~100,000, CAS No. 9003-20-7) was purchased from Central Drug House (CDH), New Delhi, India. Strontium nitrate (M.W. of 211.63, CAS No. 10042-76-9, assay min. 98.00%), silver nitrate (M.W. of 169.87, CAS No. 7761-88-8, assay min. 99.00%), sodium nitrate (M.W. of 84.99, CAS No. 7631-99-4, assay min. 98.50%), and sodium hydroxide (M.W. of 40.00, CAS No. 1310-73-2, assay 97.00–100.50%) were purchased from Himedia Chemical Suppliers, Shri Krishna Scientific, Bangalore, Karnataka, India. Double-distilled water was used throughout the experiment.

### 2.2. Synthesis of PVA

**Figure 1:**
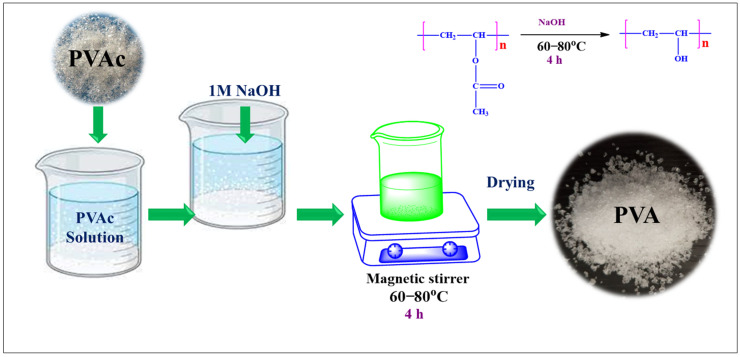
Synthesis scheme of PVA by using PVAc.

To prepare the PVA, PVAc solution (5–10%) was initially prepared by dissolving PVAc resin in distilled water. The solution was stirred continuously until PVAc dissolved completely. After ensuring complete dissolution, the hydrolysis of PVAc was carried out using 1 M NaOH solution. To the PVAc solution, 1 M NaOH solution was added slowly with constant stirring. The total volume of NaOH required depends on the degree of hydrolysis, but the NaOH used was equivalent to the PVAc concentration used. The reaction mixture was maintained at 60–80 °C using a magnetic stirrer and the solution was stirred continuously for 4–6 h. The substitution of hydroxyl groups for acetate groups caused the PVAc to undergo a conversion to PVA. The progress of the reaction was monitored by maintaining the pH and checking the viscosity of the reaction mixture. Furthermore, once the reaction was complete, acetic acid was utilized to neutralize the solution, which was later allowed to cool down at room temperature. Finally, the formed PVA was precipitated using a large amount of distilled water via a filtration setup. Later, the PVA precipitate was washed continuously to remove the residual NaOH and acetic acid. Later, PVA was allowed to dry completely using an oven at 40 °C. The synthetic scheme of PVA is shown in Figure 1.

### 2.3. Microwave Extraction of Lablab Purpureus Peel (LPE)

Lablab purpureus is one of the most widely grown crops in South Karnataka. Fresh Lablab purpureus fruit peels were collected from a local market in Bangalore, Karnataka, India. The collected peels were washed multiple times with running water and then sun-dried for 15 days. After being shade-dried for five to seven days, the peels were milled into a fine powder. The dried peels were ground into a fine powder, and 50 g of the powder was then immersed in distilled water and extracted in a microwave chemical reactor. The mixture was subjected to microwave irradiation in a 35 mL glass tube under nitrogen at 60 °C for 20 min, using a power setting of 200 W. The extraction is rich in phytochemicals such as polyphenols, flavonoids, anthocyanins, and organic acids, which serve as natural reducing and stabilizing agents.

### 2.4. Synthesis of Sr-Ag_2_O Nanoparticles and PVA/Sr-Ag_2_O (PSr-Ag_2_O) Nanocomposite Films

A total volume of 100 mL of distilled water was used as the reaction medium in a clean beaker. This was mixed with 10 mL of (0.1 M) silver nitrate, calculated based on stoichiometric requirements. Later, 2 g of Lablab purpureus peel extract was added to the reaction mixture and stirred continuously for 30 min. Subsequently, 10 mL (0.1 M) strontium nitrate solution was added dropwise to the same 100 mL mixture until a black precipitate formed. The reaction mixture was placed in a 100 mL Teflon-lined autoclave and heated to 120 °C for 4 h as part of the hydrothermal treatment. Once the product had cooled to room temperature, it was purified by washing it multiple times with ethanol and distilled water. The product was then dried overnight in an oven at 80 °C.

The PVA/Sr-Ag_2_O (PSr-Ag_2_O) nanocomposite films were prepared by mixing 2 g of PVA in 100 mL of deionized water using a magnetic stirrer to help it dissolve. Next, different amounts of Sr-Ag_2_O nanoparticles (0.01 g, 0.02 g, and 0.03 g) were added to the PVA solution to create varying weight ratios. The mixture was subjected to sonication for 30 min to ensure uniform dispersion of Sr-Ag_2_O particles within the PVA matrix. The resulting PVA/Sr-Ag_2_O solution was then poured into sterilized Petri dishes and manually rotated to distribute the solution evenly, forming a thin film layer. The films were exposed to microwave radiation in a microwave oven at a constant power of 200 W in an air atmosphere (internal heating in the range of 80–100 °C). The samples underwent cyclic microwave irradiation, with each cycle consisting of 3 min of exposure followed by cooling to room temperature to mitigate heat accumulation. This process was repeated for a total of five cycles to obtain stable and thoroughly dried PVA/Sr-Ag_2_O nanocomposite films (abbreviated as PSr-Ag_2_O). After ensuring the complete evaporation of solvent and film formation, the films were manually peeled from the Petri dishes and stored in a desiccator for further investigation. As a reference sample, a PVA film was also prepared. The summary of the composition is shown in Table 1.

### 2.5. Characterizations

Film thickness was measured using a Mitutoyo Dial Thickness Gauge (made in Kawasaki, Japan). At different locations, thickness measurements were taken and averaged. The thickness of all the nanocomposite films was found to be around 0.18 mm. The presence of Sr-Ag_2_O was verified using a UV-Vis spectrophotometer (manufactured by PG Instruments Limited, based in Luton, Bedfordshire, United Kingdom), specifically a two-beam T80 UV-Vis spectrophotometer, which captured spectra ranging from 200 to 800 nm.

The formation and presence of Sr-Ag_2_O nanoparticles in PVA films were determined by using ATR-FTIR (purchased from Prestige 21 Company, Shimadzu, Kyoto, Japan). All tests were carried out within the range of 400–4000 cm^−1^ at a resolution of 4 cm^−1^.

The mechanical properties of the prepared nanocomposite films were determined using LLOYD Instrument Universal Testing Machine (UTM, Hampshire, UK). All mechanical tests were conducted in accordance with ASTM D882-91 [38]. The film samples, which were cut to 2.5 cm × 10 cm in size (rectangular), were fastened using the extension grips of the testing apparatus. The samples were characterized to determine their tensile strength, Young’s modulus, and %Elongation at break. All tests were conducted at room temperature at a speed of 5 mm/min.

The prepared PVA film, PSr-Ag_2_O nanocomposite films, and Sr-Ag_2_O nanoparticles were subjected to morphology studies using scanning electron microscopy (SEM-FESEM, JEOL JSM-6360, Tokyo, Japan). The tests were conducted using an acceleration voltage of 5 kV. The sputter coating of conducting gold was applied to all samples before the experiment to protect them from being charged by intense electron beams. The film samples were attached to the material holder using double-sided sticky carbon tapes.

A transmission electron microscopy (TEM) micrograph was recorded using a JEM-1400, JEOL Limited (Tokyo, Japan), at an acceleration voltage of 200 kV. The nanocomposite film with a 1 mg/mL concentration solution was cast onto a carbon-coated copper microgrid with a mesh size of approximately 200 and air-dried. For the TEM analysis, Sr-Ag_2_O NPs and PSr-Ag_2_O nanocomposite films were subjected to grid preparation using Tetrahydrofuran (THF) and acetone.

The NPs and nanocomposite films synthesized Sr-Ag_2_O nanoparticles and PSr-Ag_2_O nanocomposite films were examined via an X-ray diffraction spectrometer (Raigaku D/Max-IIA, Tokyo, Japan). The radiation was produced by a Cu-Ka source that ran at 30 kV and had a wavelength of 1.5406 Å. Scan rates of 2°/min were achieved with a current of 20 mA and a 2θ range of 0–80°.

The degradation of the produced Sr-Ag_2_O NPs and PSr-Ag_2_O nanocomposite films was investigated using thermogravimetric analysis (TGA) with the aid of SDT-Q600-V20.9TA instruments (New Castle, Delaware, USA). In an inert N_2_ atmosphere, samples weighing about 6–8 mg were heated to 800 °C at a rate of 10 °C/min.

The contact angle measure was used to assess the hydrophobicity and hydrophilicity of the pristine PVA and PSr-Ag_2_O nanocomposite films. The SEO Phoenix device (Seoul, Korea) was used to measure the water contact angle. A 7 µL size drop was used for each test, which were all conducted at room temperature. Using the Phoenix contact angle software (SEO Phoenix 300), contact angle investigation was performed at nearly five different locations.

#### 2.5.1. Antimicrobial Study

Nanocomposite films made of PVA and Sr-Ag_2_O were evaluated in vitro against two strains of harmful bacteria: *E. coli* (*Escherichia coli*-ATCC 25992) and *P. aeruginosa* (*Pseudomonas aeruginosa*-NCIB 8295). To test the antimicrobial efficacy, the disk diffusion method was employed. Bacteria were introduced into a 100 mL flask of nutrient broth and incubated at 37 °C for nearly 18 h. All the organisms to be tested were carefully placed on sterile Mueller–Hinton agar plates. Dimethyl sulfoxide (DMSO) was used to dissolve the films, creating solutions with a concentration of 0.02 mg/mL. Later, infected bacterial plates were covered with 1 mm sterile disks that had been impregnated with film solutions of varying concentrations. The study included streptomycin and ampicillin as bacterial positive controls. Under the optimal temperature needed for the bacterial species, these plates were incubated at 37 °C for 24 h. The results show the average, with some variation, from all three separate tests, and we measured the antibacterial activity by looking at the average size of the areas where bacteria were stopped, measured in millimeters. Determining the well’s zone of inhibition enabled us to measure its antibacterial activity.

#### 2.5.2. Cytotoxicity

The Sr-Ag_2_O NPs and PSr-Ag_2_O nanocomposite films were subjected to cytotoxicity studies. When performing cytotoxicity analyses, it was necessary to trypsinize the cells and then aspirate their contents into a 15 mL centrifuge tube. The cells were concentrated into a pellet by spinning them in a centrifuge at 300× *g*. The cell count was controlled by suspending around 10,000 cells in 200 µL of DMEM medium. The cell suspension was applied to each 96-well microtiter plate, with approximately 200 µL of the suspension. The plates were then incubated at 37 °C in a 5% CO_2_ environment for approximately 24 h. After that time, the spent media were removed. Finally, the overall concentration was altered to 0.5 mg/mL by adding 100 μL of medium containing 10% MTT reagent to each well. The plate was then incubated at 37 °C in a 5% CO_2_ environment for 3 h. In the next step, 100 μL of DMSO solution was added to the media that had been removed, and the plate was gently shaken in a rotary shaker to mix the formazan that had formed. A microplate reader employed an absorbance spectrophotometer with two wavelengths 570 nm and 630 nm. Through the utilization of the cell line’s dose–response curve, the quantity of progression inhibition was determined after eliminating the blank and background. Subsequently, the IC_50_ was calculated by determining the concentration of the test medicine that resulted in a 50% inhibition of cell growth.

#### 2.5.3. Blood Compatibility (Haemolysis)

The haemolysis assay measured the amount of haemolysis caused by Sr-Ag_2_O NPs and PSr-Ag_2_O nanocomposite films when exposed to fresh human blood, using data obtained from 5 mL of blood from a healthy test subject. Flow cytometry was used to separate the blood cells. A slow separation process followed the mixing of leucocytes and plasma at 3000 rpm for 10 min, during which the entire mixture was kept at room temperature. The red blood cell pellets were removed from the normal physiological salt water by washing them multiple times with 0.9% sodium chloride. After 10 min of centrifugation at 3000 rpm and washing, the blood sample becomes clear. A day of incubation at 37 °C followed by soaking the 1 cm × 1 cm films in normal saline. Then, 200 μL of blood and 200 μL of incubated test samples were added and incubated for 30 min at 37 °C. After adding 4 mL of physiological saline to the specimens and allowing them to sit for 60 min, haemolysis was prevented. As both negative and positive controls, respectively, film specimens were not employed in the haemolysis of red blood cells in distilled water and sterile physiological saline. Afterwards, the supernatant’s absorbance at 540 nm was determined by centrifugation at 3000 rpm for 10 min. The following formula was used to calculate the % of haemolysis.

(1) $$\%\;of\;Haemolysis=\frac{Test\;sample\;abs-Negative\;control\;abs}{Positive\;control\;abs-Negative\;control\;abs}\times 100$$

#### 2.5.4. Cell Migration (Wound-Healing Scratch Assay)

The preparation of Sr-Ag_2_O NPs and PSr-Ag_2_O nanocomposite films was investigated for cell migration. During the wound-healing assay, a 5 mL centrifuge tube was used for aspirating the trypsinized cells. Centrifugation at 300× *g* was used to extract the cell pellet and cell counts were adjusted by using DMEM. After adding 1 mL of DMEM fluid with the cell suspension to each well of the 12-well plates, the plates were incubated at 37 °C in a 5% CO_2_ environment for 24 h, until the cells had grown into a single layer, covering the entire bottom of the well. Without changing the medium, the monolayer was carefully and slowly scratched across the well’s center using a fresh 200 μL pipette tip. While scratching across the surface of the well, the long axis of the tip should always be perpendicular to the bottom of the well. The resultant space between the two objects is thus the same as the tips outside diameter. Various types of tips allow for adjustments to be made to the gap distance. In this process, a directional scratch mark was made. To remove the detached cells, the well was gently rinsed with medium twice after scratching. The cells were washed twice with 1× PBS, and then new media were added to the well. Aspiration of the PBS was performed. Next, 1 mL of fresh medium was added to each well along with the test concentrations derived from the stock of test medicines. Before incubating the plate at 37 °C in a 5% CO_2_ environment for 24 h, photographs of the scratched monolayer were taken at various time intervals 0 h, 06 h, 12 h, and 24 h. By calibrating measurements at a 4× resolution, MagVision 4.6 Software can generate a quantitative assessment of the gap distance.

The following formula was utilized to ascertain the migration rate:

(2) $$R_{m}=\frac{\left(W_{i}-W_{t}\right)}{T}$$

where R_m_—rate of cell migration (µm/h), W_i_—initial wound width (µm), W_t_—final wound width (µm), and T—duration of migration (hours).

(3) $$\%\;of\;wound\;closure=\frac{Initial\;wound\;diameter-Final\;wound\;diameter}{Initial\;wound\;diameter}\times 100$$

## 3. Results and Discussion

### 3.1. UV–Visible Spectroscopy

**Figure 2:**
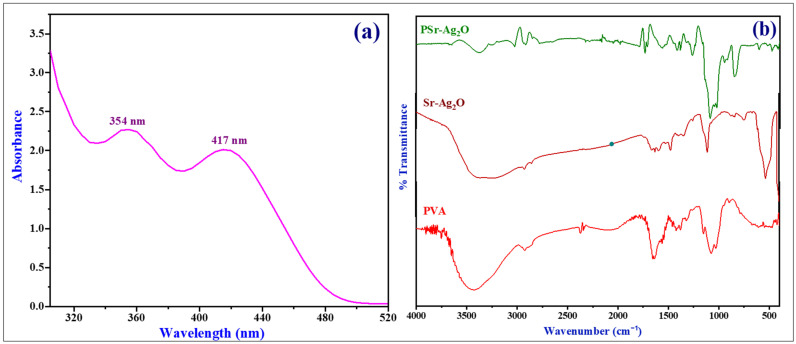
(**a**) UV–visible spectra of Sr-Ag_2_O nanoparticles; (**b**) FTIR spectra of PVA, Sr-Ag_2_O NPs, and PSr-Ag_2_O nanocomposite films.

The formation and optical properties of Sr-Ag_2_O nanocomposites were investigated using UV–Vis spectrophotometry. The absorption spectrum (Figure 2a) exhibited two prominent peaks at 354 nm and 417 nm, which are attributed to the characteristic transitions of Sr-Ag_2_O nanoparticles. The absorption band around 417 nm corresponds to the surface plasmon resonance (SPR) of Ag_2_O. In comparison, the blue-shifted peak at 354 nm indicates a modification in the band structure, likely induced by the incorporation or surface interaction of Sr^2+^ ions. The absence of any additional absorption bands in the UV region suggests the formation of a phase-pure Sr-Ag_2_O nanostructure without residual precursors or secondary phases. This is further supported by comparative literature, where silver nanoparticles typically exhibit SPR bands in the 390–470 nm range, and SrO-based nanoparticles show absorption in the 250–350 nm region [39]. The observed blue shift in our composite system can be attributed to synergistic effects, including quantum confinement resulting from reduced particle size, lattice strain, and alterations to the electronic structure due to the incorporation of Sr. Moreover, the interaction of Sr^2+^ with the Ag_2_O matrix likely leads to localized dielectric environment changes and interface effects, which further modulate the optical response. Strontium oxide nanoparticles, often produced via green synthesis, exhibit absorbance maxima in the range of 250–350 nm [39].

The results may differ, nevertheless, depending on the experimental conditions, nanoparticle composition, shape, and size. According to Ayinde et al., the absorption peaks of the silver nanoparticles were found to be most prominent in the 390–470 nm wavelength range [40,41]. In a similar vein, Jyoti et al. found that nanoparticle materials exhibiting peaks in the 410–450 nm wavelength band showed a more distinct spherical form [41]. These findings are consistent with SEM and TEM analyses, which confirm the formation of spherical, uniformly dispersed nanoparticles. The combination of structural and optical features confirms the successful green synthesis of Sr-Ag_2_O nanocomposites with tailored electronic and morphological characteristics.

### 3.2. Fourier Transform Infrared Spectroscopy (FTIR)

The FTIR spectra of PVA, Sr-Ag_2_O NPs and PSr-Ag_2_O nanocomposite films are summarized in Figure 2b. The characteristic absorption peaks for pure PVA were recorded at 3420 cm^−1^, corresponding to the –OH stretching vibration, and peaks observed at 2626 and 2861 cm^−1^ were attributed to the asymmetric and symmetric stretching of C–H alkyl groups present in the PVA. Furthermore, the transmittance peak observed at 1661 cm^−1^ corresponds to the C=O stretching of the acetate group in PVA, and the peaks at 1431 cm^−1^ were assigned due to the presence of C–H bending of CH_2_ bending and the peak observed in the range of 1080–1151 cm^−1^ is due to acetyl C–O stretching of PVA. The peak that occurred at 894 cm^−1^ corresponds to the rocking of the C–C band of PVA [42,43]. Additionally, when strontium coating is present, it may slightly change where or how strongly the Ag-O stretching occurs, suggesting that new bonds might be forming or the surface is being altered. This signal could indicate the formation of new bonds or surface modifications.

**Figure 3:**
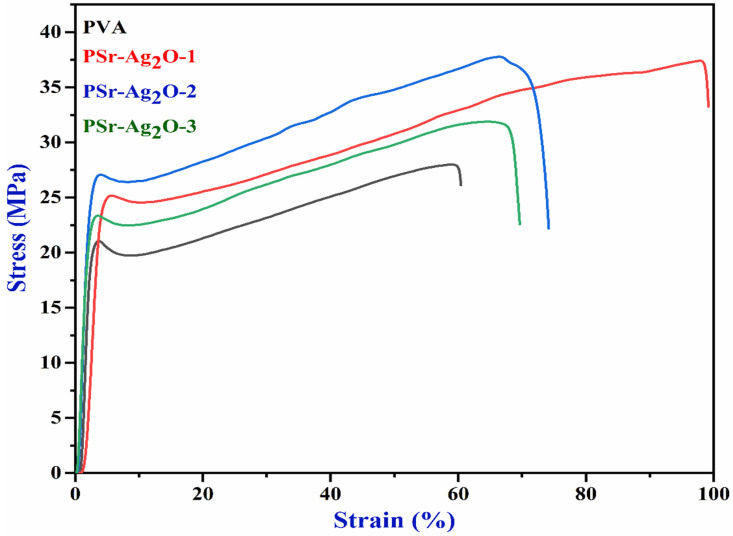
Stress–strain curve of pure PVA and PSr-Ag_2_O nanocomposite films.

It is quite probable that the hydroxyl groups (–OH) on the surface of the nanoparticle or absorbed water molecules are responsible for the O–H stretching vibration, as indicated by the peak at 3380 cm^−1^. The peak at 474 cm^−1^, which is usually used to identify metal oxides, is probably caused by the bending vibration of the metal–oxygen (Sr-O and Ag-O) bonds in the Sr-Ag_2_O NPs [44,45]. Perhaps related to the existence of Sr-O and Ag-O bands, additional strong and noticeable peaks occurred in the range of 841, 599, and 474 cm^−1^ and have evolved. The stretching vibrations observed at 1093 cm^−1^ could be attributed to Sr-O and Ag-O, indicating the presence of strontium-coated silver oxide nanoparticles in PVA nanocomposite films. The data presented in Figure 3 suggest that the addition of Sr-Ag_2_O nanoparticles to the PVA matrix results in an increase in the stretching vibrations of O–H, C–H, and C=O groups. The addition of Sr-Ag_2_O nanoparticles to the PVA polymer chain has resulted in changes in the stretching vibrations at 3420, 1080, and 894 cm^−1^ in the PVA matrix. The stretching vibrations observed at 1400–1600 cm^−1^ correspond to the C–H bond of CH_2_, which becomes broken with the addition of Sr-Ag_2_O NPs, leading to increased band intensities. Sr-Ag_2_O NPs have created new stretching bands at 940–936 cm^−1^ and 856 cm^−1^, indicating that these nanoparticles are present in the PVA film.

### 3.3. Mechanical Properties

A wound dressing should ideally be strong and flexible enough to cover the wound and fit it in its proper location [46]. Nanocomposite films must possess specific mechanical properties to be employed in wound-healing applications. Table 2 illustrates the mechanical properties of the PVA and PSr-Ag_2_O nanocomposite films, and Figure 3 shows the stress–strain curve. The results demonstrated that pure PVA film showed tensile strength (T_s_) of 27.988 MPa, a Young’s modulus (Y_m_) of 979.277 MPa, and a %Elongation at break (%E_b_) of 60.424%. The lower weight of Sr-Ag_2_O nanoparticles (PSr-Ag_2_O-1) increased the T_s_ of PVA film from 27 to 37 MPa, and Y_m_ increased from 979.277 to 1204.066 MPa. Meanwhile, %E_b_ decreased from 394.32 to 99.21% in the PSr-Ag_2_O-1, indicating a decrease in the flexibility of nanocomposite films, which could be due to the restriction of the ductile flow of the PVA polymeric chains. Similar results were observed in polyvinyl alcohol-/lignin-blended films [47]. The reduction in elongation at break indicates an increase in the rigidity of the films with the incorporation of nanoparticles into the PVA film. As Sr-Ag_2_O nanoparticles increased in the PVA matrix, a general decline in Ts and %E_b_ was observed, due to the reduced flexibility and ductility of the nanocomposite films. A slight increase in Y_m_ for PSr-Ag_2_O-2 compared to PSr-Ag_2_O-1 was observed, likely due to improved filler–matrix interaction at this concentration. The reinforcement effect caused a temporary rise in stiffness, then declined at higher concentrations.

Moreover, as the weight of the Sr-Ag_2_O nanoparticles increased in the PVA film, reduced T_s_, Y_m_, and %E_b_ were observed, as shown in Table 2. These results were in line with the work of other authors [48,49]. As expected, adding Sr-Ag_2_O nanoparticles to the PVA film results in an enhancement in the T_s_ and Y_m_. This improvement may be attributed to the strong interaction and compatibility between the PVA polymer chains and Sr-Ag_2_O nanoparticles. As shown by the stress–strain curve, PSr-Ag_2_O nanocomposite films become flexible than pure PVA and are not completely brittle. Such quality is essential to maintaining the skin’s natural elasticity. More significantly, the value of this nanocomposite film is comparable to that of skin, making it useful for wound applications [50].

### 3.4. Morphology

**Figure 4:**
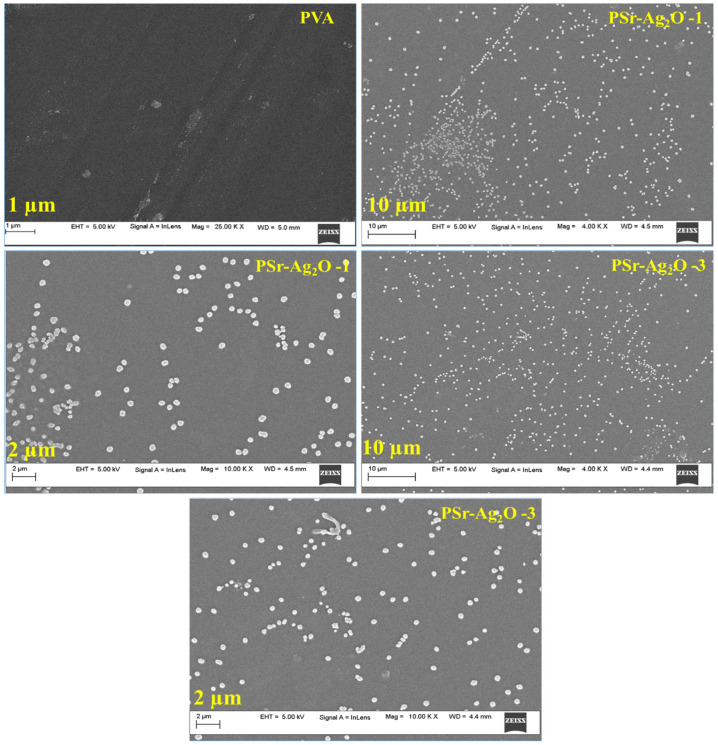
SEM images of PVA and PSr-Ag_2_O nanocomposite films at different magnifications.

SEM was used to conduct a comprehensive examination of the microstructure of the built nanocomposites (Figure 4). SEM analysis showed that the surface structure of the PVA matrix changed noticeably after adding Sr-Ag_2_O nanoparticles. For the morphological study, we have selected lower and higher concentrations (PSr-Ag_2_O-1 and PSr-Ag_2_O-3) to investigate the influence of Sr-Ag_2_O on the PVA film. The authors intentionally excluded the PSr-Ag_2_O-2 (second film), as the intention was to study the effect of the nanoparticle concentration within the PVA matrix by selecting two representative formulations (lower and higher concentrations (PSr-Ag_2_O-1 and PSr-Ag_2_O-3)). Across all samples, the nanofillers appeared to be uniformly embedded within the polymer matrix, with no signs of aggregation or phase separation, indicating successful and homogeneous dispersion. At varying magnification levels, the nanocomposite films retained a continuous and defect-free morphology. Pristine PVA exhibited a characteristically smooth and featureless surface, consistent with its typical polymeric structure. However, when Sr-Ag_2_O was added, the films became noticeably rougher and exhibited round bumps, indicating the presence of tiny particles.

The spherical features were spread out evenly and did not clump together, indicating that the nanomaterial was well mixed into the polymer structure. Similar results were reported by Abdu Saeed et al., who prepared flexible nanocomposite films of ZnO and polyvinyl chloride/poly(N-vinyl carbazole) polymers. The addition of ZnO NPs results in rough composite films due to the presence of small white granules. Similar observations have been noted in PVA/ZnO films, where the dense region of the PVA/ZnO film, upon the addition of ZnO nanoparticles (NPs), became rough. Numerous microscale bright spots were observed in this area, representing sections of ZnO NPs dispersed within the PVA [51].

**Figure 5:**
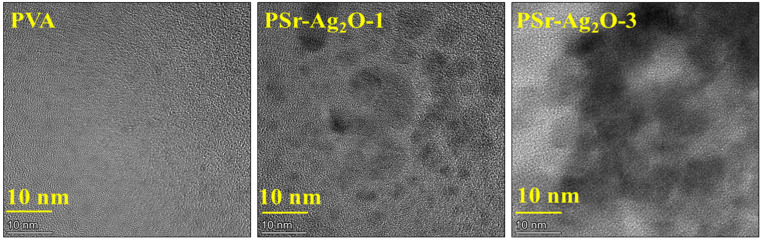
TEM micrographs of pure PVA and PSr-Ag_2_O nanocomposite films.

To further probe the nanostructural arrangement, TEM was employed. TEM provided high-resolution confirmation of the observations made via SEM, clearly visualizing discrete, nanoscale spherical Sr-Ag_2_O particles within the PVA matrix shown in Figure 5. The particles, predominantly within the nanometer range, were well dispersed without forming clusters, reaffirming the homogeneous distribution inferred from SEM imaging. The tiny spherical shapes seen in TEM match the shapes found in SEM, supporting the idea that Sr-Ag_2_O particles are evenly spread throughout the nanocomposite. The absence of large clumps or distinct areas in both SEM and TEM tests indicates that the manufacturing process was carefully controlled. According to the morphological investigations, the PVA films loaded with Sr-Ag_2_O had nanoparticles that were evenly dispersed throughout the matrix. However, when the concentration increased, a slight clustering of the nanoparticles was noticed [52].

The inclusion of Sr-Ag_2_O not only modifies the smooth surface profile of the neat PVA but also contributes to the development of a well-organized internal architecture. This additional information from both surface and internal imaging techniques demonstrates how the addition of Sr-Ag_2_O significantly affects the shape and structure of the PSr-Ag_2_O nanocomposite films. The even distribution and fine arrangement of the fillers are likely to affect the nanocomposite’s physical, chemical, and possibly functional properties.

### 3.5. XRD Studies

**Figure 6:**
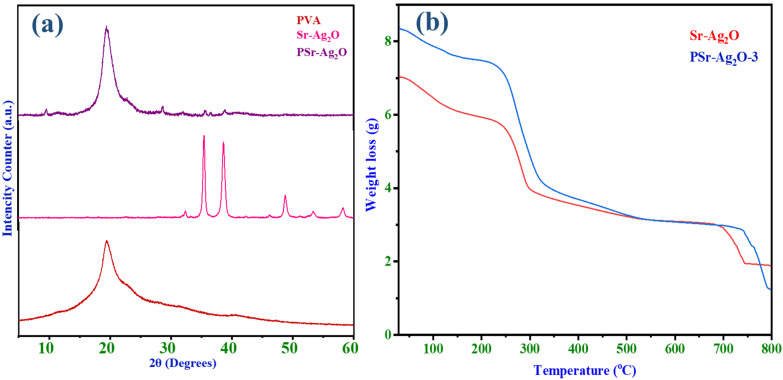
(**a**) X-ray diffractogram of pure PVA, Sr-Ag_2_O NPs and PSr-Ag_2_O nanocomposite films; (**b**) thermogravimetric images of Sr-Ag_2_O NPs and PSr-Ag_2_O-3 nanocomposite films.

Figure 6a shows the powder XRD patterns of pristine PVA, strontium-coated silver oxide (Sr-Ag_2_O), and the combined material PSr-Ag_2_O. The authors have selected PVA, Sr-Ag_2_O and PSr-Ag_2_O-3. Our objective was to compare the crystallinity and phase behavior of the PVA film with the incorporation of Sr-Ag_2_O. For better clarity, we have used Sr-Ag_2_O nanoparticle XRD to help us understand the effects of Sr-Ag_2_O nanoparticle content on the polymer matrix. As PSr-Ag_2_O-3 demonstrated more prominent diffraction peaks and enhanced crystallinity features, this combination was subsequently selected for the biological and functional analysis. In Figure 6a, the clear peak seen at 2θ = 21° for pure PVA shows that it has a partly crystalline structure [53], which is due to the strong hydrogen bonds between the polymer chains. After adding Sr to Ag_2_O, the main crystal structure stays the same, as seen in Figure 6a, where diffraction peaks appear at 2θ values of 33.02°, 38.28°, 55.26°, and 65.86°. According to the standard JCPDS data (Card No. 75-1532), these peaks correspond to the (1 1 1), (2 0 0), (2 2 0), and (3 1 1) planes of cubic AgO. The absence of extra diffraction signals indicates that Sr is evenly mixed into the Ag_2_O structure without creating any separate crystal forms. The XRD pattern confirms the formation of the PSr-Ag_2_O nanocomposite, which is evidenced by the characteristic peaks that appeared in both PVA and Sr-Ag_2_O. Sr-Ag_2_O nanoparticles insertion leads to a decrease in the intensity owing to the interaction between the PVA and Sr-Ag_2_O nanoparticles. The addition of Sr-Ag_2_O to the PVA is indicated by the appearance of a sharp peak at 2θ = 35.47° and 38.89°. A similar result was reported by Menazea AA et al. [54]. The reduced intensity of Sr-Ag_2_O peaks is likely due to its lower content within the polymer matrix.

### 3.6. Thermogravimetric Analysis

Thermogravimetric analysis was used to examine the thermal stability of the PVA, Sr-Ag_2_O, and PSr-Ag_2_O-3 nanocomposite films, and the results are shown in Figure 6b. In our previous publication, TGA was used to characterize the PVA film; as reported, three distinct steps of deterioration were visible in the TGA curve of PVA [55]. Sr-Ag_2_O nanoparticles were added to study the thermal behavior of the synthesized nanoparticles individually, and PSr-Ag_2_O-3 nanocomposite films as formulations with the highest nanoparticle concentration were selected, reflecting the most pronounced interaction among the nanoparticles and polymer matrix, thus providing clarity with regard to the composites’ enhancement. Initial degradation occurred at 38–147 °C and was attributed to the removal of absorbed moisture. A second incidence of degradation was observed at 147–277 °C, with maximum weight loss attributed to the thermal degradation of the PVA chain. The third step of degradation was observed in the range of 277–586 °C, which was deemed to be related to the release of by-products produced during the second degradation step [55]. It is evident that, under the analytical circumstances, the strontium-coated silver oxide nanoparticles decompose below 744 °C and show four-step degradation. Initial weight loss occurred at temperatures ranging from 30 to 145 °C due to the removal of absorbed moisture and excess water molecules. The second degradation step was observed at 239–303 °C, where Sr-Ag_2_O nanoparticles remained disconnected from the PVA chains, allowing their degradation to occur at lower temperatures. When the Sr-Ag_2_O nanoparticles were evenly dispersed and fully bonded to the PVA matrix, a noticeable improvement in thermal stability was observed, with decomposition occurring at 744 °C.

In the case of the PSr-Ag_2_O-3 nanocomposite, the primary decomposition occurred between 239 and 684 °C, with a corresponding 85% weight loss. The results show that PVA films containing Sr-Ag_2_O nanoparticles were more thermally stable than the reference PVA film. The enhanced thermal stability resulting from the incorporation of Sr-Ag_2_O nanoparticles into PVA may be attributed to the interaction between the nano Sr-Ag_2_O and the hydroxyl groups of the polymer matrix.

### 3.7. Contact Angle Analysis

**Figure 7:**
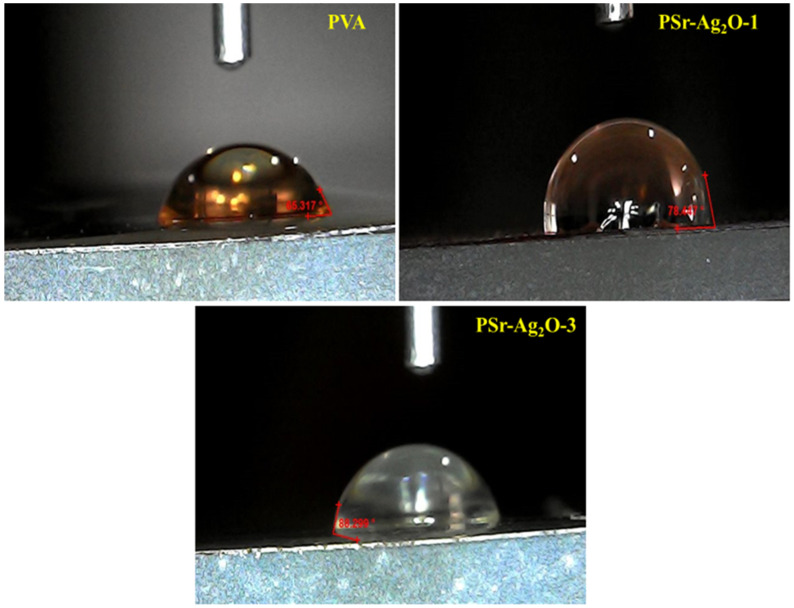
Images of the contact angle of PVA and PSr-Ag_2_O nanocomposite films.

The investigations of the hydrophilic and hydrophobic behavior of the prepared PVA and PSr-Ag_2_O nanocomposite films were determined by using contact angle measurements in a controlled laboratory at 25 °C. The contact measurement images are shown in Figure 7 and Table 3. PVA shows intrinsic hydrophilicity with a contact angle of 65.3°, which could be attributed to the presence of –OH groups in the PVA backbone. On decorating Sr-Ag_2_O nanoparticles, the contact angles of PSr-Ag_2_O-1 and PSr-Ag_2_O-3 nanocomposite films appeared to be 78.61 and 88.29, which could be attributed to the decreased hydrophilicity of PVA-based nanocomposites. This could be attributed to the involvement of hydroxyl moieties on the Sr-Ag_2_O nanoparticles. Furthermore, plant-mediated Sr-Ag_2_O nanoparticles may reduce the mobility of polymer chains in nanocomposite films by creating free spaces within the PVA matrix. This results in a reduction in hydrophilicity and an increase in hydrophobicity in the PSr-Ag_2_O nanocomposite films. The SEM study supports the idea that the greater surface roughness of the resulting films might amplify this phenomenon. The increased surface roughness restricts the liquid’s wetting ability to interact with and penetrate the surface of the nanocomposite [56].

### 3.8. Antimicrobial Study

**Figure 8:**
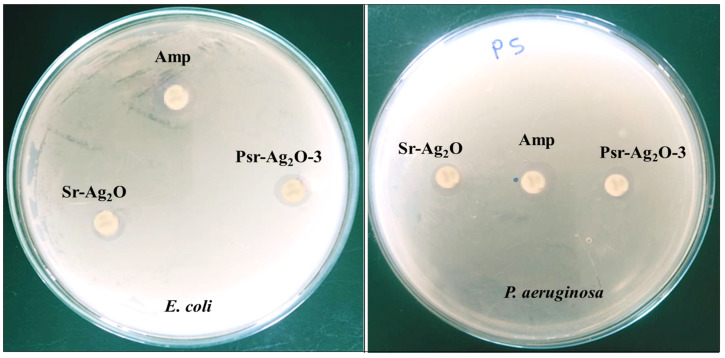
Antimicrobial images of Sr-Ag_2_O NPs and PSr-Ag_2_O nanocomposite films.

The NPs and nanocomposite films were subjected to antimicrobial investigations, and the results are summarized in Figure 8 and Table 4. Nonetheless, LP phytochemicals have sparked scientific interest and are being studied for their potential biological properties, including antioxidant, anti-inflammatory, antifungal, antibacterial, antiviral, and anticancer activities [57,58]. The antimicrobial efficacy of Sr-Ag_2_O and PSr-Ag_2_O was evaluated using *E. coli* and *P. aeruginosa*, as both are clinically significant, Gram-negative bacterial pathogens that are frequently associated with chronic wounds and nosocomial infections. Testing against these bacteria provides a strong indication of the antimicrobial efficacy of the produced nanocomposite films. The efficacy of the prepared films was compared with that of the standard drug Ampicillin. The antibacterial tests showed that Sr-Ag_2_O and PSr-Ag_2_O-3 nanocomposite films were effective against three harmful bacteria. The prepared Sr-Ag_2_O nanoparticles were most effective against *E. coli*, showing a large area of effectiveness (11.3 ± 0.4), but were less effective against *P. aeruginosa*, with a smaller area of effectiveness (8.7 ± 0.7). Among the prepared nanocomposite films, the highest zone of inhibition was observed for PSr-Ag_2_O-3 (12.7 ± 0.8 mm *E. coli*), and moderate activity was noted for 11.8 ± 0.5 *P. aeruginosa*.

The differences in the zone of inhibition can be attributed to the morphological variations between each organism. The antimicrobial activity of Sr-Ag_2_O and PSr-Ag_2_O-3 occurs because the positively charged molecules interact with the negatively charged bacterial membranes, causing damage and cell death. Typically, bacteria have a phospholipid membrane that is responsible for the structural components of lipopolysaccharides. The presence of hydroxyl groups or phenolic compounds disrupts the cytoplasmic membrane, lowering the pH gradient and creating a pathway for protons to exchange. Some microorganisms possess an outer layer of peptidoglycan, which makes their membrane more fragile. Cell death results from the release of adenosine triphosphate due to disturbance of the proton motive force and electron transport.

### 3.9. Cytotoxicity Study

**Figure 9:**
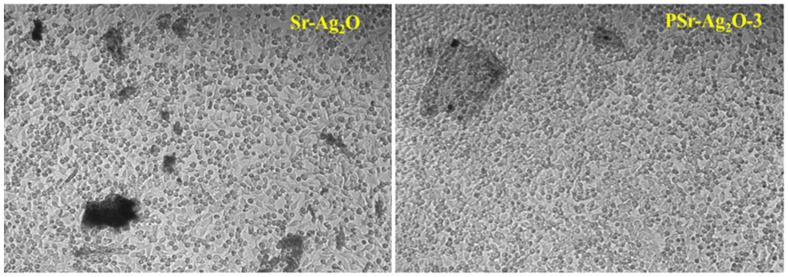
Cytotoxicity images of Sr-Ag_2_O NPs and PSr-Ag_2_O-3 nanocomposite films.

**Figure 10:**
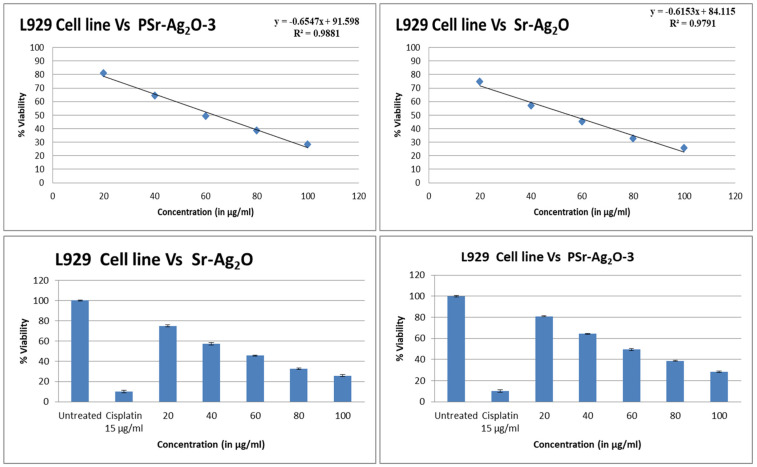
Cell viability of Sr-Ag_2_O NPs and PSr-Ag_2_O-3 nanocomposite films.

Since the material’s biocompatibility is crucial for its potential medical applications, the cytotoxicity of the samples must be taken into consideration. Using the L929 fibroblast cell line, we conducted cell viability tests to prove that Sr-Ag_2_O NPs and PSr-Ag_2_O nanocomposite films are cytocompatible [59,60,61,62]. Extensive testing was performed on samples with high concentrations, including PSr-Ag_2_O-3 nanocomposite films and Sr-Ag_2_O, to ensure their non-toxicity and the survival of cells. The nanocomposite films were tested for cell proliferation using the MTT assay at 24 h to determine their potential for wound-healing applications. Table 5 presents the results of the tests on cell survival and toxicity for Sr-Ag_2_O NPs and PSr-Ag_2_O-3 nanocomposite films, while Figure 9 and Figure 10 display the corresponding images. The accepted view is that PVA is both biocompatible and cytotoxically inert. Due to its low toxicity and high biocompatibility, it has found widespread application in the biomedical industry [63]. Previous publications have detailed cytotoxicity studies of PVA nanocomposites reinforced with green-synthesized CuONPs. After 24 h, the IC_50_ values for the untreated and standard medication Cisplatin cell lines were around 100 and 10.21, respectively. The nanocomposite films and produced Sr-Ag_2_O NPs were evaluated using the same reference values [55]. Apart from the standard drug cisplatin, the results confirm that overall, the cells remain viable after treatment with the respective substance at a particular concentration. The Sr-Ag_2_O NPs and Psr-Ag_2_O-3 nanocomposite films at lower concentrations (20 and 40 µg/mL) exhibit relatively greater cell survivability (74.92% and 80.88% for 20 µg/mL, and 57.22% and 64.40% for 40 µg/mL), as shown in Table 5. This implies that these concentrations have a less significant cytotoxicity influence on the L929 cell line. Moving forward, increased concentration (80 and 100 µg/mL) resulted in a more pronounced cytotoxic influence, with cell viability dropping to 32.65% and 25.68% for Sr-Ag_2_O and Psr-Ag_2_O-3, respectively, and to 38.62% and 28.31% for Sr-Ag_2_O and Psr-Ag_2_O-3, respectively. This indicates that a concentration above 80 µg/mL significantly influences the viability of the L929 cell line, suggesting that cytotoxicity is concentration-dependent.

Additionally, after 24 h of testing with the L929 cell line, the amounts needed to reduce cell survival by half were found to be 55.44 µg/mL for Sr-Ag_2_O and 63.53 µg/mL for Psr-Ag_2_O-3. The results demonstrate that concentrations of 55.44 µg/mL and 63.53 µg/mL are needed to reduce cell viability by 50% when subjected to the materials being tested. The improved cell survival with Psr-Ag_2_O-3 may be attributed to changes in the nanocomposite film’s biological properties resulting from various chemical effects. The lowest amounts of Sr-Ag_2_O NPs and Psr-Ag_2_O-3 nanocomposite films were found to be safe for the cell lines that were studied. Cell viability was also improved in PVA films that contained Sr-Ag_2_O.

### 3.10. Assay Performed to Study Blood Compatibility (Haemolysis)

Among the most essential, sought-after characteristics in biomedical polymeric matrices or nanocomposite films is blood compatibility [64]. When designing biomedical products, it is essential to consider hemolysis whenever the material or substance comes into contact with blood. In this context, prepared films were subjected to the haemolysis study. The study’s findings are shown in Table 5. The breakdown of red blood cells, or hemolysis, is the usual procedure for releasing hemoglobin into the blood and other bodily fluids.

The % of hemolysis value observed for Sr-Ag_2_O NPs and Psr-Ag_2_O-3 nanocomposite films were 38.56% and 44.75%. This suggests that exposure of Sr-Ag_2_O NPs and Psr-Ag_2_O-3 nanocomposite films exhibited an acceptable decrease in the degree of haemolysis for the tested film samples. Similarly, Sr-Ag_2_O nanoparticles showed lower hemolytic activity, whereas PSr-Ag_2_O nanocomposite films showed slightly increased (44.75%) haemolysis, indicating that approximately 44.75% of the blood cells were lysed. None of the films were hemolytic, demonstrating their compatibility and biocompatibility. The extremely hydrophilic properties of the polymer matrix components may explain the reduced haemolysis rate [65,66].

### 3.11. Cell Migration Study

**Figure 11:**
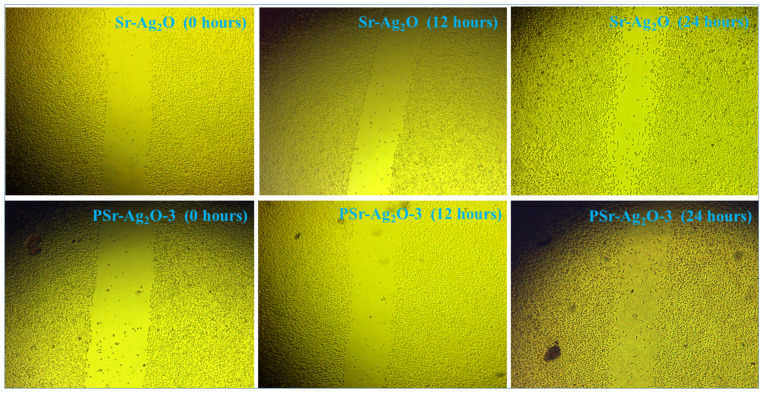
Images summarize the wound-healing scratch assay of Sr-Ag_2_O NPs and PSr-Ag_2_O-3 nanocomposite films.

Cell migration and biocompatibility of the Sr-Ag_2_O NPs and PSr-Ag_2_O-3 nanocomposite films were assessed to investigate the nanocomposite films’ applicability towards host tissue (wound-healing scratch assay). The scratch test was used to determine the efficacy of proliferation and migration of cells in regenerating skin structure during in vitro wound healing [67]. Figure 11 presents the photographs of scratched areas taken at 0, 12, and 24 h for Sr-Ag_2_O NPs and PSr-Ag_2_O-3 nanocomposite films. Table 6 presents the results of the cell migration and wound closure study after 24 h. The scratch (wound) created at 0 h is shown by the bright vertical area in each picture. Cell migration into the scratched area between 0 and 24 h is a sign that the wound is closing. In our previous publication, we found that the untreated group exhibited cell migration values of 6.70 and 5.45 at 12 and 24 h, while the conventional Ascorbic acid therapy group exhibited values of 45.40 and 22.10. The produced PSr-Ag_2_O nanocomposite film and Sr-Ag_2_O nanoparticles were tested for cell migration values in comparison to the same untreated group and the conventional Ascorbic acid drug [55].

Figure 11 shows that Sr-Ag_2_O nanoparticles had the highest cell migration after 12 h. The PSr-Ag_2_O-3 film also exhibited appreciable cell migration activity, showing nearly 19.95% cell migration compared to the standard drug. The quantitative analysis confirmed that the addition of Sr-Ag_2_O-3 in PVA showed no reduction in cell migration activity, but rather increased the cell migration. Several intrinsic characteristics of polymeric biomaterials, including material chemistry, molecular weight, solubility, implant morphology, shape, and structure, as well as their hydrophilicity/hydrophobicity and degradation mechanism, can impact their biological compatibility [68]. After 24 h, it was noted that more than 30% and 32% of the gaps were filled, showing that the PSr-Ag_2_O nanocomposite films made with Sr-Ag_2_O are suitable for use as biomaterials. For both Sr-Ag_2_O and PSr-Ag_2_O nanocomposites, an evident decrease in the breadth of the scratch area was observed at 12 h, and a significant reduction was noted at 24 h compared to the normal medication and untreated samples. The greater rate of wound closure seen in PSr-Ag_2_O-3 when compared to Sr-Ag_2_O alone suggests that the polymer matrix and Sr-Ag_2_O NPs work in concert to promote migration of cells and growth. The presence of Sr-Ag_2_O nanoparticles favored the adhesion and spreading of cells, which triggered the reduction in the gaps.

This visual proof illustrates how the PSr-Ag_2_O-3 nanocomposite film can facilitate wound healing. In vivo wound-healing experiments are anticipated to be conducted as part of our future research to validate the efficacy of these wound-dressing films on actual wounds. Furthermore, the molecular process of induced cell migration may be better understood through investigations of gene and protein expression.

## 4. Conclusions

The development of nanocomposite films embedded with nanoparticles prepared using plant extracts is attracting significant interest due to these films’ potential applications in areas such as wound healing and biomedicine. This work aimed to create Sr-Ag_2_O nanoparticle-embedded PVA nanocomposite films through solution casting. Various characterization methods were used to examine the impact of Sr-Ag_2_O nanoparticles on the structural, mechanical, morphological, antimicrobial, and bioactive properties of the PVA nanocomposite films. UV–visible and FTIR tests confirmed that Sr-Ag_2_O nanoparticles formed and were present in the PVA film. Mechanical, morphological, water contact angle, and thermal studies confirmed the enhanced mechanical and thermal properties, reduced hydrophilicity, and improved the compatibility of PVA nanocomposite (PSr-Ag_2_O) films. Crystalline Sr-Ag_2_O phases were identified in the PVA film, as shown by the XRD patterns. The antimicrobial, cytotoxicity, and hemolysis results demonstrated that Sr-Ag_2_O NPs and PSr-Ag_2_O nanocomposite films exhibited notable antimicrobial activity, lower hemolytic activity, and non-toxic behavior at lower concentrations. The wound-healing scratch assay showed that adding Sr-Ag_2_O-3 to PVA did not hinder cell migration; on the contrary, it enhanced it. The presence of Sr-Ag_2_O nanoparticles promoted cell adhesion and spreading, which contributed to reducing gaps. Overall, the results indicate that the produced nanocomposite films could have promising applications in wound healing.

## Figures and Tables

**Table 1:** Chemical make-up of nanocomposite films. (The total weight of the film was 2 g).

Sample Name	Thickness (mm)	PVA (g)	Sr-Ag_2_O (g)
PVA	0.09	2	0
PSr-Ag_2_O-1	0.08	1.98	0.02
PSr-Ag_2_O-2	0.07	1.96	0.04
PSr-Ag_2_O-3	0.09	1.94	0.06

**Table 2:** Mechanical properties of PVA and PSr-Ag_2_O nanocomposite films.

Sample Name	Tensile Strength (T_s_)	Young’s Modulus (Y_m_)	% Elongation at Break (E_b_)
PVA	27.98 ± 1.2	979.27 ± 20	394.32 ± 5.1
PSr-Ag_2_O-1	37.40 ± 1.5	970.69 ± 25	99.21 ± 4.8
PSr-Ag_2_O-2	36.76 ± 1.3	1204.06 ± 18	74.17 ± 6.0
PSr-Ag_2_O-3	31.88 ± 1.4	1093.98 ± 22	69.65 ± 4.5

**Table 3:** Contact angle values of PVA and PSr-Ag_2_O nanocomposites.

Film Sample	Contact Angle (°)
PVA	65.37 ± 2.2
PSr-Ag_2_O-1	78.46 ± 1.7
PSr-Ag_2_O-3	88.29 ± 2.3

**Table 4:** In vivo antibacterial activity of Sr-Ag_2_O NPs and PSr-Ag_2_O-3 nanocomposite films.

SampleName	Inhibition Zone (Diameter of Growth in mm)	
	*E. coli*	*P. aeruginosa*
Sr-Ag_2_O	11.3 ± 0.4	8.7 ± 0.7
PSr-Ag_2_O-3	12.7 ± 0.8	11.8 ± 0.5
Ampicillin	20.2 ± 0.6	21.3 ± 0.6

*E. coli* ATCC 25992 and *P*. *aeruginosa* NCIB 8295. Standard antibiotic disks: ampicillin (10 mg); negative result. Values are the mean ± standard error of the mean (SEM). All incubations were performed in triplicate.

**Table 5:** The IC_50_ values were recorded for the L929 cell line for about 24 h of treatment and Haemolysis of Sr-Ag_2_O NPs and PSr-Ag_2_O-3 nanocomposite films.

Sample Name	L929 Cell Line IC_50_ (in µg/mL) 24 h	Haemolysis (%)
Sr-Ag_2_O	55.44	38.56 ± 0.0775
PSr-Ag_2_O-3	63.53	44.75 ± 0.0052

For IC_50_ values outside of the experimental concentration range taken, the values provided above are only expected values; re-testing may be required at the higher/lower concentration range for confirmation.

**Table 6:** Cell migration study of Sr-Ag_2_O NPs and PSr-Ag_2_O-3 nanocomposite films.

Sample Name	Duration (h)	Cell Migration (µm)	% of Wound Closure (24 h)
Sr-Ag_2_O (15 µg)	12	22.48	30.82
	24	17.49	
PSr-Ag_2_O-3 (15 µg)	12	19.95	32.87
	24	7.70	

## Data Availability

The original contributions presented in this study are included in the article. Further inquiries can be directed to the corresponding authors.
